# Article writing

**DOI:** 10.4103/0971-3026.69345

**Published:** 2010-08

**Authors:** Bhavin Jankharia

**Affiliations:** Editor-in-Chief, The Indian Journal of Radiology and Imaging “F”, 1^st^ Floor, Bhaveshwar Vihar, 383, Sardar V P Road, Mumbai - 400 004, India. E-mail: editor@ijri.org

A couple of months ago, I was asked to speak on ‘How to Write a Scientific Paper,’ at a local state meeting. I made a list of points to be covered, but the focus in the end was on what not to do, rather than on what to do.

Essentially,

1. Please do not write case reports – more than 80% of the case reports will be rejected.
2. Please do not plagiarize. Write in your own language.
3. Please get the article checked by someone well-versed in English writing, especially if English is not your first language.
4. Please follow the ‘Guidelines for Authors’ strictly.

I am not even starting with the scientific content, statistical analysis, and other issues that are required to create a good article. What I am talking about here is very basic stuff that applies to anything composed in ink on paper or nowadays on a computer.

And yet, more than 80% of all articles submitted do not even adhere to these basic rules. Earlier we used to spend a lot of time and effort correcting these articles, getting the spelling, grammar, and language checked, and hand-holding the authors. However, this has led to our inability to focus on the bigger issues and is bogging us down. The two-fold increase in submissions, after the journal has become indexed, is also not helping. As a result, the IJRI, over the last quarter, has decided that if an article is badly written, irrespective of its merit, it will be rejected, with one chance given to the author to get the article rewritten by someone who knows how to write.

The current issue carries an article by Shantanu Ghosh, from the Behavioral and Cognitive Science Laboratory of the Indian Institute of Technology, New Delhi. Ordinarily, this article would be considered too technical for a general purpose journal such as the IJRI, but the reason for including this article titled, ‘Functional Mapping of Language Networks in the Normal Brain using a Word-Association Task,’ is simply to showcase what good research is all about. I wanted to bring out the fact that it is possible to do good work and write it, while sticking to the guidelines. Although the subject matter is not a core clinical topic, it serves as a guide to what is possible.

The issue also carries case reports on MRI of the brain in cerebral malaria, acute reversible toxic encephalopathy, and metronidazole toxicity, all essentially showing how the brain reacts to acute and subacute insults.

We continue our focus on local interventional radiology, with an article on MRI-guided breast biopsy from a center that has done the first few ones in our country.

As the journal continues to grow in stature, it is now necessary to expand the number of active editors working with the journal. Over the next two-to-three months, we will have speciality-based internal editors, who will also become more hands-on in the actual evaluation of the articles and the creation of quarterly issues. If you believe you have the qualifications for this or know someone who does, please let me know. Hopefully, in the years to come, this will create a talent pool of radiologists who are well-versed in editing journals and who will be able to carry the torch forward.

